# Tetracycline (TC) removal from wastewater with activated carbon (AC) obtained from waste grape marc: activated carbon characterization and adsorption mechanism

**DOI:** 10.1007/s11356-024-33493-6

**Published:** 2024-05-01

**Authors:** Semanur Sağlam, Feride N. Türk, Hasan Arslanoğlu

**Affiliations:** 1 Department of Chemical Engineering, Faculty of Engineering, Canakkale Onsekiz Mart University, Çanakkale, Turkey; 2 Central Research Laboratory Application and Research Center, Çankırı Karatekin University, Çankırı, Turkey

**Keywords:** Tetracycline, Antibiotic removal, Activated carbon, Water pollution

## Abstract

In this study, activated carbons were obtained from grape marc for tetracycline removal from wastewater. Activated carbons were obtained by subjecting them to pyrolysis at 300, 500, and 700 °C, respectively, and the effect of pyrolysis temperature on activated carbons was investigated. The physicochemical and surface properties of the activated carbons were evaluated by SEM, FTIR, XRD, elemental analysis, N_2_ adsorption/desorption isothermal, thermal gravimetric (TG) and derivative thermogravimetric (DTG), and BET surface area analysis. When the BET surface areas were examined, it was found that 4.25 m^2^/g for activated carbon was produced at 300 °C, 44.23 m^2^/g for activated carbon obtained at 500 °C and 44.23 m^2^/g at 700 °C, which showed that the BET surface areas increased with increasing pyrolysis temperatures. The pore volumes of the synthesized activated carbons were 0.0037 cm^3^/g, 0.023 cm^3^/g, and 0.305 cm^3^/g for pyrolysis temperatures of 300, 500, and 700 °C, respectively, while the average pore size was found to be 8.02 nm, 9.45 nm, and 10.29 nm, respectively. A better adsorption capacity was observed due to the decrease in oxygen-rich functional groups with increasing pyrolysis temperature. It was observed that the activated carbon obtained from grape skins can easily treat hazardous wastewater containing tetracycline due to its high carbon content and surface functional groups. It was also shown that the activated carbon synthesized in this study has a higher pore volume despite its low surface area compared to the studies in the literature. Thanks to the high pore volume and surface active groups, a successful tetracycline removal was achieved.

**Graphical Abstract:**

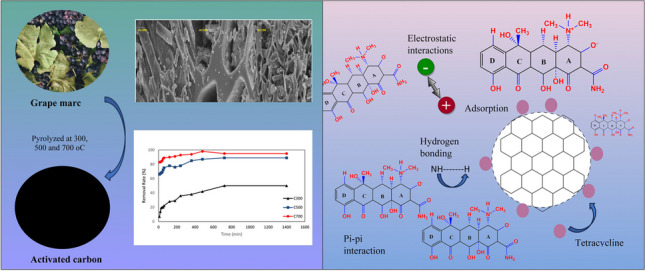

## Introductıon

Antibiotics are widely used in animal husbandry (Dehmani and Abouarnadasse 2020), health (Rezaee et al. 2022), and agriculture. They are preferred because they increase microbial resistance in health, increase crop productivity in agriculture, and support growth in livestock. Only between 2000 and 2015, a 39% increase in antibiotic consumption in the health sector was reported ( Yu et al. 2020). Antibiotics, which are noted for their remarkable properties, are widely used in some countries, such as the USA and Korea, to stimulate growth. Annual drug consumption is reported to be between 100 and 200 thousand tons globally according to 2003 data (Daghrir and Drogui 2013). In addition to the health sector, antibiotics are also popular in the livestock sector and are preferred both to prevent microbial infections and to support growth in animal farms (Vu et al. 2010). However, since antibiotics are absorbed slowly by most humans and animals, 30–90% of their main components end up in water, leading to increased resistance to antibiotics in microorganisms. In the Andriamanohiarisoamanana study, Andriamanohiarisoamanana reported that 71–90% of antibiotics are excreted from the body due to the low digestibility of antibiotics in animals. In 2013 alone, 131,109 tons of antibiotics were consumed, of which 91,776 to 117,998 tons were excreted. According to research, a 35% increase in antibiotic consumption is expected between 2013 and 2030 (Andriamanohiarisoamanana et al. 2020; Klein et al. 2018). High levels of antibiotic content were found especially in animals. For example, it was reported that 10.82 mg/kg tetracycline, 291.9 mg/g fluoroquinolone, and 41.3 mg/kg sulfonamide antibiotics were found in pig manure, which is animal waste (Cela-Dablanca et al. 2020; Delgadillo-Mirquez et al. 2022; Gaballah et al. 2024). Antibiotics enter the environment mainly through hospital wastewater, industrial production, animal manure, and irrigated farmland (Zeng et al. 2024) . These decontaminated wastewaters are highly hazardous to the environment due to their dangerous effects such as providing microbial resistance and being bioaccumulative.

Tetracycline (TC), one of the most widely used antibiotics, is used to treat microbial infections in humans and animals. Discovered in the 1940s, TC is known for being a broad-spectrum antibiotic that inhibits protein synthesis (Ae et al. 2008; Coyne et al. 1994; Daghrir and Drogui 2013). It is also preferred because it is a broad-spectrum antibiotic that shows activity against a large number of gram-positive and gram-negative bacteria, atypical organisms, and protozoan parasites (Daghrir and Drogui 2013). However, antibiotics such as TC are difficult to recover from wastewater because they cause microorganisms known as “superbugs” to produce antibiotic-resistant genes (R. Wang et al. 2016). On the other hand, the World Health Organization has reported that exposure to pharmaceutical pathogens in the environment poses a serious health threat, as they easily form stable compounds with ions such as Cs and Ca + 2 (Tian et al. 2016).

TC has ionizable functional groups such as dimethyl amino group (C-4), phenolic diketone group (C-10:C-11:C-12), and tri carbonylamide group (C-1:C-2:C-3) (Antón-Herrero et al. 2018; Gu and Karthikeyan 2005). These functional groups vary according to different pKa values, which is an important parameter in removal. The fact that it has cationic and anionic species under acidic, moderately acidic to neutral and alkaline conditions is important for determining in which pH range the most effective removal will be achieved in the recovery from wastewater. On the other hand, it is necessary to examine the surface functional groups in order to understand the TC removal mechanism. TC functional groups are generally known to be tri carbonylamide at 3.3, phenolic diketone at 7.68, and dimethylamine at 9.69 (Vu et al. 2010).

Although TC is found in wastewater at as little as 1 ng/L, its presence in wastewater is very serious for living organisms. For this reason, researchers have used various wastewater treatment techniques such as biodegradation (Gómez-Pacheco et al. 2011), adsorption technology (Kanmaz et al. 2023), chemical oxidation (Jeong et al. 2010), and photocatalytic degradation and photoelectrocatalytic degradation (Zeng et al. 2024) to treat TC-containing wastewater (Kanmaz et al. 2023). Adsorption technology is one of the most preferred techniques due to its cost-effectiveness, easy availability, and high adsorption capacity (Sağlam et al. 2024). On the other hand, conventional wastewater treatment technologies based on activated sludge are problematic because they exhibit bacterial resistance. Adsorption technologies with effective and efficient adsorption capacity are used. There are many types of adsorbents in adsorption technology such as zeolite (Pasimli 2013), graphene, activated carbon (Arslanoğlu 2019), black coal, graphene oxide (Gao et al. 2012), biochar (Shao et al. 2024), magnetic activated carbon (Yang et al. 2024), composite adsorbents (Kubra et al. 2023), and metal–organic frameworks (Sağlam et al. 2023). Among the adsorbents, especially composite-based adsorbents are studied by many researchers (Awual et al. 2024; Awual 2019; Awual et al. 2014; Hossain et al. 2024; Li et al. 2023; Rasee et al. 2023; Sheikh et al. 2023; Waliullah et al. 2023). Composite materials attract attention with their high surface area, reusability, and adsorption capacity. In addition to its amazing features, it also has production costs and production challenges. On the other hand, researchers have turned to activated carbons with high adsorption capacity and surface areas, especially in recent years, in order to reduce the cost. However, the cost of commercial activated carbons is also high. Fort his reason, in recent years, the studies carried out within the scope of waste management and sustainability, especially biochar produced from lignocellulosic sources, have attracted great interest. Biochar is obtained as a result of the thermochemical decomposition of materials containing oxygenated functional groups and aromatic groups by pyrolysis at high temperatures (300–900 °C), in the presence of very little oxygen or in the absence of oxygen and in the presence of non-condensable gases such as nitrogen gas (Chandra and Bhattacharya 2019). In the literature, Auricularia auricula wastes (Dai et al. 2020b), rice straw (Dai et al. 2020a), sugar cane bagasse (Cai et al. 2019), grapefruit peel (Yu et al. 2020), tea waste (Li et al. 2021), and grape marc (Onat et al. 2023). In the studies carried out, tetracycline removal was realized and lignocellulosic sources were utilized. In this context, considering the examples in the literature, it was observed that activated carbons provide an effective adsorption capacity for tetracycline removal. Therefore, it can be said that activated carbon is a potential solution for antibiotic wastewater treatment.

Tetracycline is one of the most widely used antibiotics. Introduced between 1960 and 1970 as an antiviral drug, tetracycline is used very frequently, especially in the health sector, with its strong aspects such as its ability to prevent viral diseases and organ damage caused by the virus. So much so that when we look at the current situation, it is thought that this antibiotic, which is claimed to have given positive results with its use in the early stage of the COVID-19 disease, which shook the whole world, emerged in 2019 and has lasted until today, will be preferred in the coming centuries. However, the damage that such a powerful antibiotic causes to our living ecosystem by mixing into wastewater also raises concerns. As a result of tetracycline consumption, a certain amount of it enters wastewater and increases antibiotic resistance in the environment where it is found, and this increase in microbial resistance causes us to encounter much stronger viruses. In this study, it was aimed to obtain activated carbon from grape marc, a lignocellulosic source. In the literature, bio-activated carbon production from grape marc has been realized before. Onat et al. (2023) reported that 46.37 mg/g tetracycline adsorption was realized with activated carbon obtained from a mixture of grape marc and vinasse (Onat et al. 2023). In another study, grape marc was used in a study conducted to investigate electricity generation performance (Taskan 2021). In other studies using grape pomace, evaluation of the physicochemical properties of the biological adsorbent was obtained from grape pomace (Perez-Ameneiro et al. 2015), C(VI) reduction from wastewater (Arslanoğlu et al. 2019), adsorption of A07 dyestuff (Bourahla et al. 2023), antioxidant activity (Negro et al. 2003), and extraction of phenolic compounds (Bonilla et al. 1999).

When the literature was examined, many other studies using grape marc were found, but none of them carried out a study on tetracycline removal. In this context, we think that our study is unique. Activated carbon was synthesized by the one-step pyrolysis method because it is both cost-effective and easy to obtain. In this study, the adsorption capacity of tetracycline adsorption capacity of activated carbons derived from grape tinder was investigated. The activated carbons obtained at different temperatures (300, 500, and 700 °C) were named AC300, AC500, and AC700. It was determined that the obtained activated carbons provided an effective adsorption/desorption thanks to the electrostatic interactions between surface functional groups and tetracycline. SEM, XRD, DTA, and TGA analyses of the obtained activated carbons were performed. In addition, BET surface area analysis, which is very important in adsorption, was performed. Adsorption capacity was analyzed by adsorption isotherms and kinetics.

## Materıal methods

### Preparation of adsorbents

**Fig. 1:**
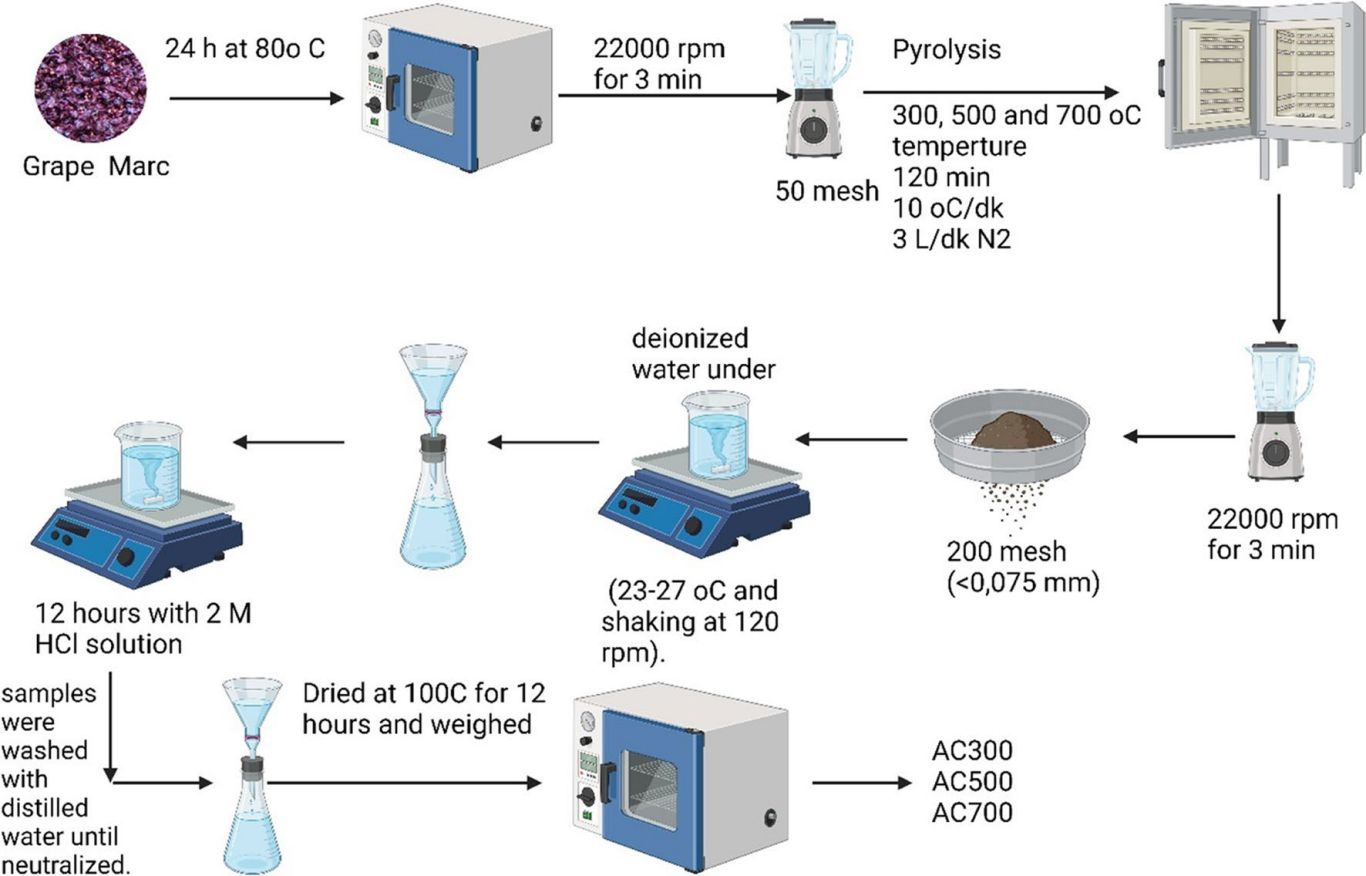
Activated carbon production

The grape marc was blended in a blender at 22000 rpm for 3 min with a particle size of 50 mesh. For pyrolysis, the samples were placed in the oven at room temperature, and 100 ml/min nitrogen gas was passed for 10 min. The pyrolysis process was carried out for 120 min in the oven brought to a certain temperature (300 °C, 500 °C, and 700 °C). Pyrolysis products were ground and sieved through a 200 mesh (< 0.075 mm sieve). The samples were subjected to extraction with deionized water under standardized conditions (23–27 °C with shaking at 120 rpm). The solid samples remaining after extraction were mixed with 2 M HCl solution for 12 h with shaking, and the washed samples were washed with distilled water to neutrality. The final solid sample was dried at 100 °C for 12 h and weighed. The solid product obtained after filtration is called activated carbon. After drying, activated carbon is labeled AC300, AC500, and AC700 according to the pyrolysis temperature of 300, 500, and 700 °C (Fig. 1).

### Characterization of adsorbents

After obtaining ACs from grape marc at different pyrolysis temperatures, the samples were analyzed by scanning electron microscopy (SEM) to describe the surface morphology at an accelerating voltage of 5.0 kV. The FTIR spectra of pyrolyzed grape marc as raw material were recorded in the wavenumber range of 400–4000 cm^−1^ and were examined for the samples belonging to the functional groups. FTIR analyses were obtained with a Perkin Elmer Model Spectrum (USA). After passing the prepared ACs, they were analyzed by X-ray diffractometer **(**XRD). The XRD was performed to determine the crystallinity of possible substances on ACs. The elemental analyzer (Elemantar Vario) was used for the elemental analysis of adsorbents. The specific analyzer (Micromeritics) was used to evaluate the surface are, porosity, and pore volume, which have a significant influence on the adsorption capacity of AC300, AC500, and AC700.

### Adsorption batch experiments

**Fig. 2:**
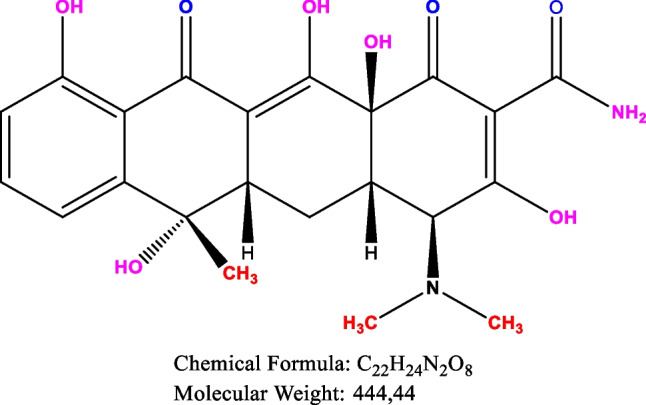
Tetracycline chemical formula (Wang et al. 2016)

The adsorption batch experiments were carried out for the adsorption capacity of ACs on TC. In the experiments, the initial concentration (10 mg L^−1^), the effects of solution pH, and the reaction contact time, which have a significant effect on adsorption, were also investigated. To a 50-mL Erlenmeyer flask, a small amount of AC was added, and 25 mL of TC was added at a certain concentration, oscillated at about 25 ℃ and 120 rpm after adsorption for a certain period, pre-filtered all suspensions through a 0.45-μm membrane, and determined the concentration of TC in the filtrate at a wavelength of 357 nm using a UV spectrophotometer (UV1200, Shimadzu) (Fig. 2). All experiments were repeated twice. A blank experiment was performed at the same time in parallel. In order to understand the adsorption effect of TC on the obtained AC, the obtained adsorption data were analyzed using kinetic models and isotherm models and interpreted with the literature.

1 $$\mathrm{Removal\;rate }\left(\mathrm{\%}\right): \mathrm{R }\left(\mathrm{\%}\right)=\frac{{{\mathrm{C}}}_{0}-{{\mathrm{C}}}_{{\mathrm{e}}}}{{{\mathrm{C}}}_{0}}\times 100$$

2 $$\mathrm{Adsorption\;capacity\;}({\mathrm{mg}}/{\mathrm{g}}): {q}_{e}=\frac{\left({{\mathrm{C}}}_{0}-{{\mathrm{C}}}_{{\mathrm{e}}}\right)V}{{\mathrm{m}}}$$

In the above equations, C_0_ (mg/L) and C_e_ (mg/L) represent the initial and equilibrium concentrations of TC, respectively, V (L) is the volume of the solution, and m (g) is the mass of the adsorbent.

Pseudo-first-order kinetic model (PFO):

3 $${\mathrm{log}}\left({{\mathrm{q}}}_{{\mathrm{e}}}-{{\mathrm{q}}}_{{\mathrm{t}}}\right)=\mathrm{log }{{\mathrm{q}}}_{{\mathrm{e}}}-\frac{{{\mathrm{k}}}_{1,{\mathrm{ad}}}}{2.303}{\mathrm{t}}$$

Pseudo-second-order kinetic model(PSO):

4 $${{\mathrm{q}}}_{{\mathrm{t}}}=\frac{t}{\frac{1}{{{\mathrm{k}}}_{2,{\mathrm{ad}}}{{{\mathrm{q}}}_{{\mathrm{e}}}}^{2}}+\frac{t}{{{\mathrm{q}}}_{{\mathrm{e}}}}}$$

In the equation, q_t_ (mg/g) represents the amount of adsorption on TC at time t (h), and k_1_ (h^−1^) and k_2_ [g/(mg·h)] represent the adsorption equilibrium rate constants for the PFO and PSO models, respectively.

Langmuir model:

5 $${{\mathrm{q}}}_{{\mathrm{e}}}=\frac{{{\mathrm{q}}}_{{\mathrm{m}}}{{\mathrm{K}}}_{{\mathrm{L}}}{{\mathrm{C}}}_{{\mathrm{e}}}}{1+{{\mathrm{K}}}_{{\mathrm{L}}}{{\mathrm{C}}}_{{\mathrm{e}}}}$$

Freundlich model:

6 $${{\mathrm{q}}}_{{\mathrm{e}}}={{{\mathrm{K}}}_{{\mathrm{F}}}{{\mathrm{C}}}_{{\mathrm{e}}}}^{{~}^{1}\!\left/ \!{~}_{{\mathrm{n}}}\right.}$$

Dimensionless constant separation factor (R_L_):

7 $${{\mathrm{R}}}_{{\mathrm{L}}}={~}^{1}\!\left/ \!{~}_{(1+{{\mathrm{K}}}_{{\mathrm{L}}}{{\mathrm{C}}}_{0})}\right.$$

### Characterization of activated carbon

**Fig. 3:**
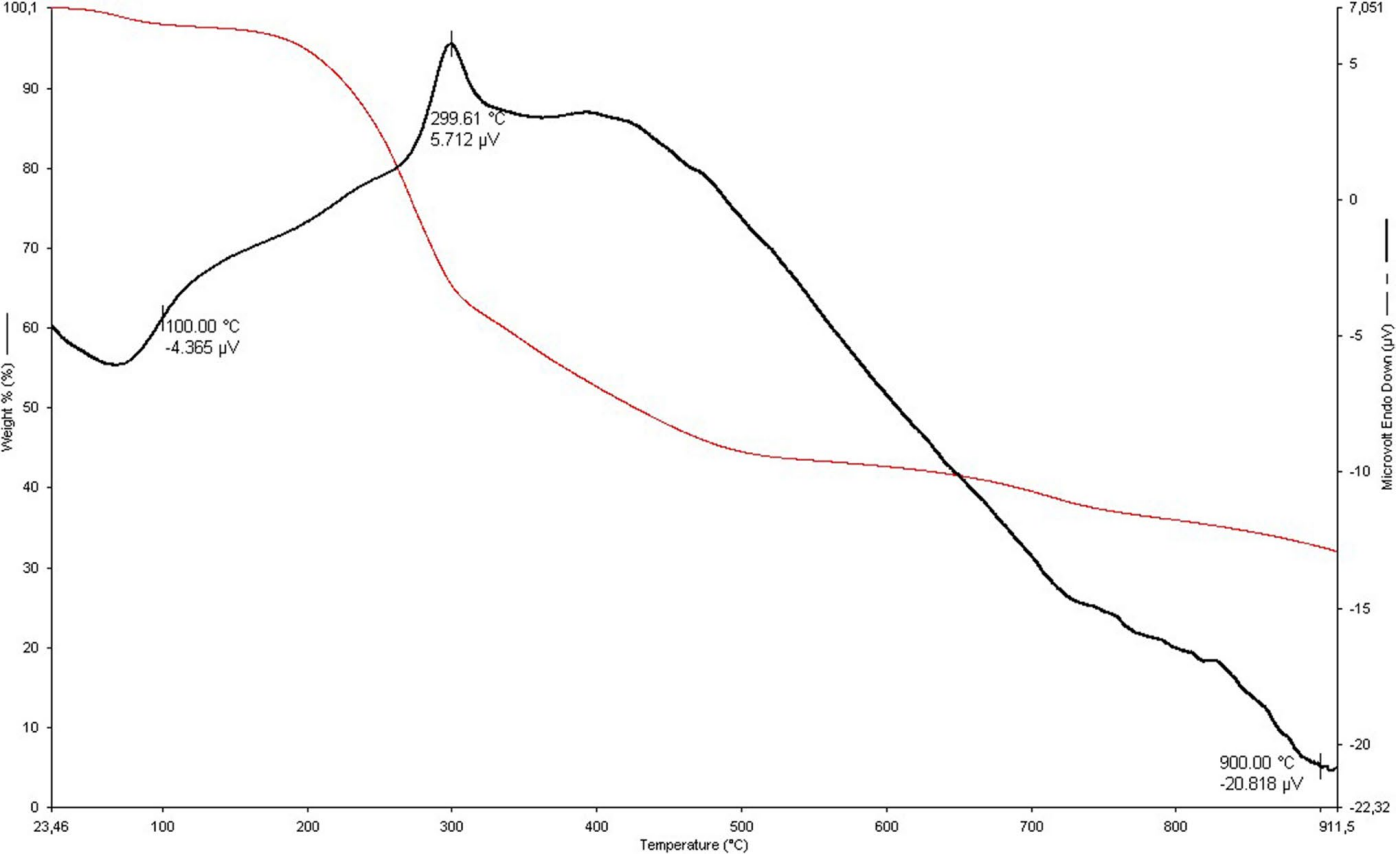
TG and DTG curves of grape marc during pyrolysis

The thermal gravimetric (TG) and derivative thermo gravimetric (DTG) curves of spent grape marc during pyrolysis are shown in Fig. 3. The rises in pyrolysis temperature show that grape marc is gradually caused weightless, which indicates that it affected the residual quality of grape march by temperature increasing between 30 and 800 °C. In the first stage of TGA analysis, the moisture of the biomass is removed between 30 and 100 °C (Arslanoğlu 2021). At an increase in the pyrolysis temperature from 100 to 200 °C, char is less weightless (4%), and pyrolysis at these temperatures is called the pre-pyrolysis stage. Similar results were observed in the study by Charmas et al. (2018), and they reported that they removed water from the material at 200 °C (Charmas et al. 2018). The results also show an increase in weight loss (6%) due to the evaporation of the water mass in the sample by heating. This is due to the high water and residual solvents in the sample. Temperatures between 200 and 300 °C are in the pyrolysis stage. In this temperature range, the weight loss reached 37%. At 200–250 °C, the cellulose has started a dehydration reaction. At temperatures between 200 and 250 °C, the cellulose started a dehydration reaction. When the reaction temperature reaches 280–340 °C, depolymerization of cellulose takes place and hemicellulose starts to break down. DTG curve reached the highest peak at 319.1 °C, which is when the weight loss is highest. After 332 °C, the main part of the material undergoes extensive pyrolysis and carbonization begins. At 635 °C, the residual mass was 18%. When the temperature is increased and pyrolysis is continued, carbonization and graphitization polymerization of the material continues. It also performs the thermal decomposition of inorganic ash.

**Fig. 4:**
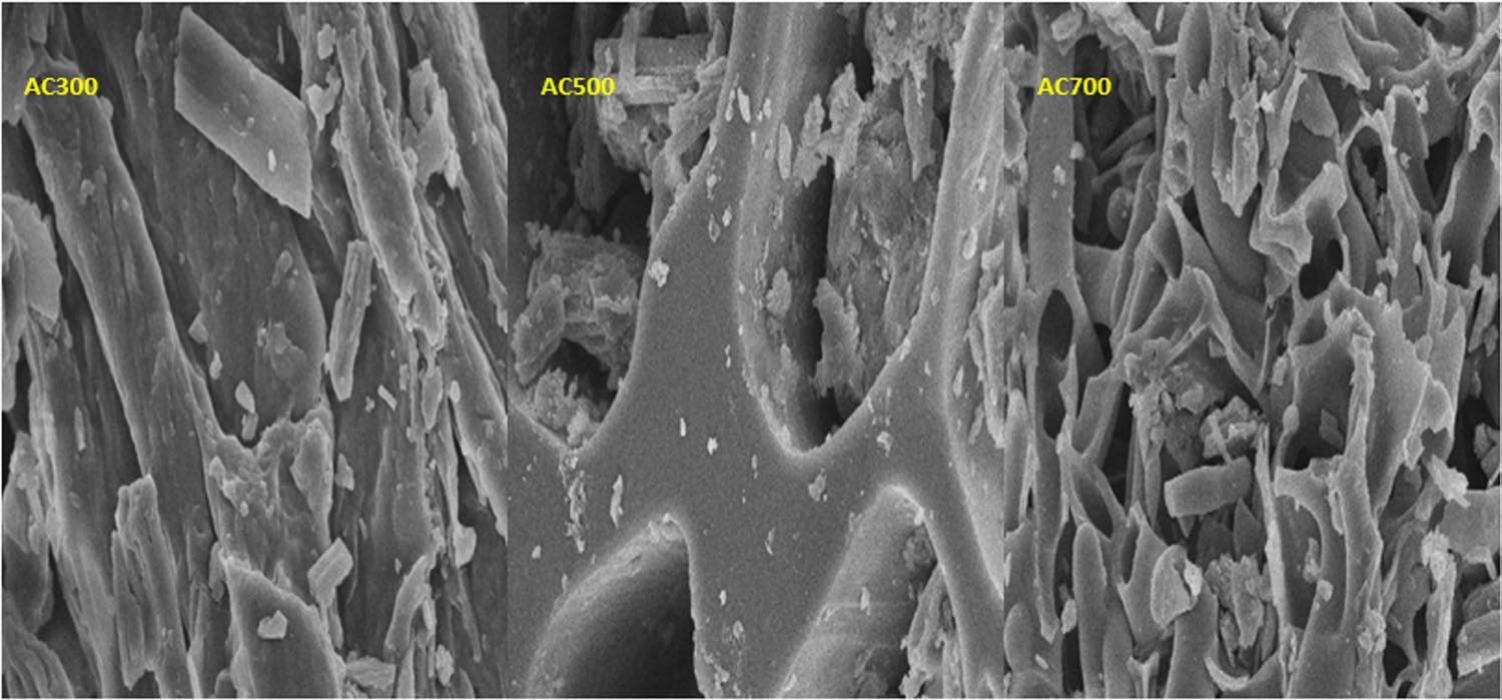
Scanning electron microscope image of activated carbons

Scanning electron microscopy analysis was carried out to characterize the porous surface structure and form of activated carbons formed at different applied temperatures and activating agent ratios. SEM images of the Grape marc sample and activated carbons produced under different experimental parameters are given in Fig. 4. As it is known, the gradual removal of different volatile compounds from the raw material during the pyrolysis process depends on the temperature increase. The temperature in the pyrolysis process affects the particle size and form of the solid material. With increasing temperature, an increase in pore size and ratio is generally observed, while a decrease in cell wall thickness is observed. Although the cell walls of activated carbons formed at low-temperature values (300 °C) are thick and covered with tar agglomerates, the cell walls of activated carbons formed at higher temperature values (500 °C, 700 °C) have a more porous and more amorphous structure, as it appears to be. In addition, the cell walls of activated carbons produced at high temperatures have become thinner and more brittle. According to the images taken from the SEM microscope, it can be said that the porosity of activated carbon increases as the pyrolysis temperature increases. Scanning electron microscopy (SEM) results of AC obtained from grape marc are given in Fig. 4. When the SEM images obtained are examined, it is seen that the particles of activated carbons are porous and consist of irregular sizes and shapes. Grape marc has different pore sizes and shapes due to various inorganic components in its structure. These differences are eliminated by the carbonization and activation process. These irregular- and different-sized pores on the surface of activated carbons contribute to the surface area, thereby increasing the adsorption capacity and improving efficiency. Marzbali et al. (2016) reported that there were too many cracks on the surface of the activated carbon they obtained in their study and that the surface was damaged as a result of the dehydration of the catalyzing agent. However, they reported that these voids increased the efficiency of TC adsorption because they provided more active sites (Marzbali et al. 2016). The results of this study show that different pyrolysis temperatures affect the surface of ACs. The surface of AC300 is partially rough and the curves are not uniform. There are no visible pores and protrusions can be seen on the surface. This is because pyrolysis and carbonization are incomplete due to the low pyrolysis temperature of AC300. This also shows that the coal is not completely carbonized. Compared to AC300, AC500 has more prominent pore structures and particle sizes are more pronounced. There are some open tube shapes on the surface of the AC700. This is due to the release of volatiles and carbon enrichment of the material.

Elemental analysis determines the proportion of C, H, and N in the sample (Rizwan et al. 2020). Table 1 shows the elemental composition of AC300, AC500, and AC700. The results show that increasing pyrolysis the temperature from 300 to 700 °C reduces oxygen (O) content and hydrogen content. There was also an increase in nitrogen (N) content from 0.94 to 1.02 and carbon content (C) from 43.58 to 50.57. The ratio of C and N components increased, while the ratio of O and H decreased. This is because grape marc undergoes corrospoding. The decrease in the N ratio varies between 1.06 and 1.61% in the literature as in this study (Yue et al. 2017). Usually during pyrolysis, nitrogen is removed as ammonia (NH_3_) and nitrogen monoxide (NO). Therefore, there is a small change in the nitrogen content. However, this change in nitrogen content does not show a significant difference after pyrolysis (Rizwan et al. 2020). The results obtained in this study show that the H and O content is correlated with the presence of functional groups. The presence of organic groups affects TC adsorption. The H/C ratio represents the aromatization and carbonization of activated carbon, and the smaller it is, the stronger the aromaticity. Aromaticity and carbonization increase at increasing temperatures. Compared to AC300, AC500, and AC700, its carbonization is the lowest because of low-temperature pyrolysis. AC300 contains non-pyrolyzed organic components such as fatty acids, lignin, and cellulose. AC300, AC500, and AC700 H/C ratios are 0.101, 0.044, and 0.30, respectively. The O/C ratio is related to the hydrophilicity of the material. The higher this ratio, the more hydrophilic it is, as the O content decreases with increasing pyrolysis temperature. The H/C ratio decreased gradually as the pyrolysis temperature increased. It was found 0.698, 0.287, and 0.229 for AC300, AC500, and AC700, respectively (Table 1). This showed that hydrophobicity increased with increasing pyrolysis temperatures. (O + N)/C represents the polarity of the AC, which is consistent with the H/C trend. With increasing pyrolysis temperature, alkyl aliphatic compounds in the waste of grape marc react to form aromatic groups and polar functional groups decrease. The H/C of AC decreases with increasing pyrolysis temperature. At the same time, the O/C and (O + N)/C ratio also decreases.

**Table 1:** Elemental composition of ACs

Activated carbon	Elemental composition				Ash	Atomic ratio		
	C	H	N	O	(%)	H/C	O/C	(O + N)/C
AC300	42.55	4.38	0.94	32.44	20.66	0.101	0.698	0.720
AC500	48.67	2.23	1.18	15.27	32.60	0.044	0.287	0.311
AC700	51.63	1.47	1.01	12.63	35.29	0.030	0.229	0.250

In a study on the adsorption of malachite green by AC prepared from grape marc at different pyrolysis temperatures, it was found that the increase in temperature resulted in the release of H, O, and N from the carbon chain to form H_2_O and volatile N containing substances, thus reducing the functional groups on the surface in the AC. In a study on the adsorption efficiency of TC by aerobic granular sludge-based AC, it was found that the oxygen-containing functional groups (OCFG) of AC can form hydrogen bonds with TC. In addition, UV radiation can increase the OCFG of AC, thus promoting the adsorption of benzene on AC. It has also been shown that NaOH modification results in more OCFG on the AC surface, which can form hydrogen bonds with hydroxyl and amino groups on TC, thus enhancing the adsorption effect. Therefore, the surface functional groups of AC form hydrogen bonds with TC and thus promote the adsorption of TC.

**Fig. 5:**
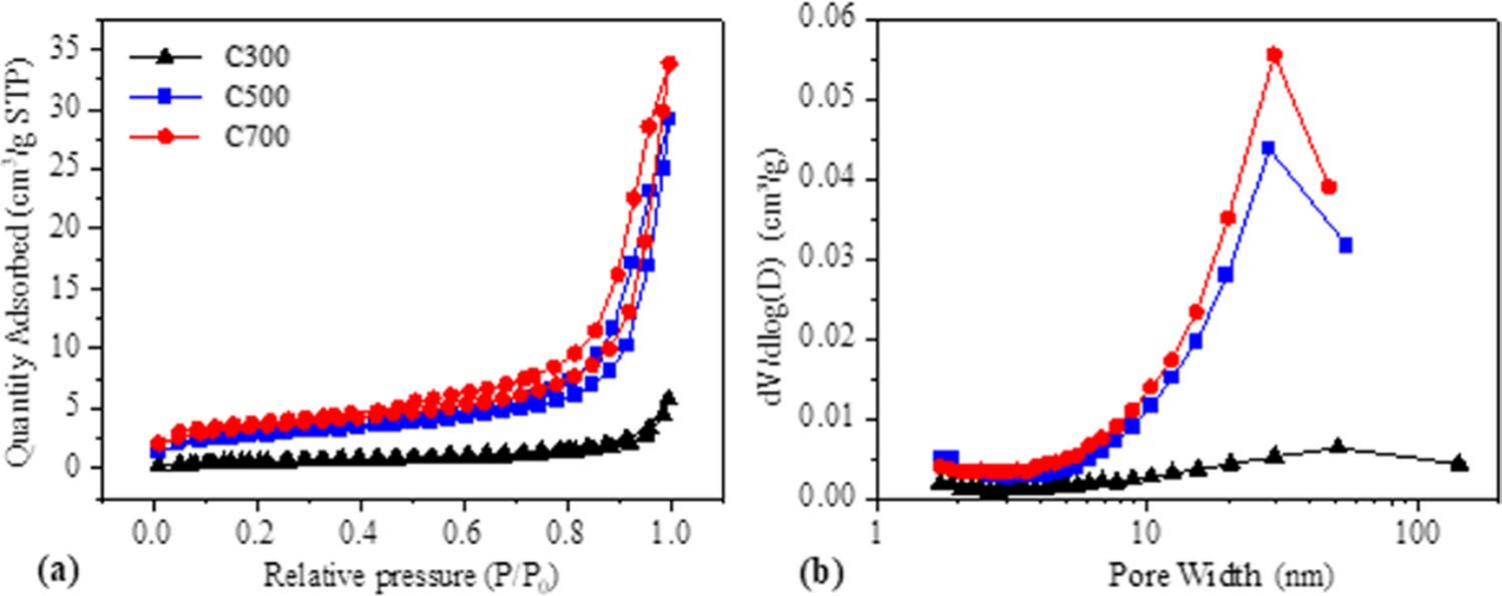
N_2_ adsorption–desorption isotherms (**a**) and pore size distribution (**b**) of ACs

Figure 4 shows the N2 adsorption/desorption isothermal and pore size distributions of AC300, AC500, and AC700. The sorption and desorption isotherms of the prepared samples at all temperatures showed different type IV curve characteristics with H1 hysteresis rings. The surface areas of all samples are BET surface areas calculated by the multi-point method from the nitrogen adsorption data. Nitrogen adsorption/desorption isotherm data of all activated carbon are given in Fig. 5. Adsorption isotherms, in which the heat of adsorption of the first layer is greater than the heat of condensation and the capillary condensation is less, are similar to this curve. The condensation of liquids in microscopic cracks and pores on the surface is called capillary adsorption. The monolayer capacity can be approximately read from the extension of the linear portion of the isotherm after the point. Cheng et al (2022) performed N2 adsorption/desorption measurements and observed that nitrogen adsorption gradually increased in microporous materials (Cheng et al. 2022). In another study, Arslanoğlu (2019) reported that the adsorbent they produced as a result of the data obtained in N2 adsorption/desorption isotherms had a mesoporous structure and adsorption occurred in a single layer (Arslanoglu 2019). When the BET surface areas were also examined in this study, an increase in the pore volume and size was observed along with the wall surface areas as the temperature increased. This was also observed in another study. Salman et al., stated that the specific surface area of the mesoporous adsorbent they made was 539.11 while it had a pore volume of 0.73 and 403.19 m^2^/g and 0.43 cm^3^/g. In this respect, the results obtained in the studies are similar (Salman et al. 2023a).

Based on the BET formula, SBET can be calculated by the N_2_ sorption and desorption isotherm. Table 2 shows that the SBET of the sample increases slightly with increasing temperature. The SBET of AC300, AC500, and AC700 are 4.25 m^2^/g, 25.94 m^2^/g, and 44.23 m^2^/g, respectively.

**Table 2:** The data of the porous structure of ACs

Sample	BET specific surface area (m^2^/g)	Pore volume (cm^3^/g)	Average pore size (nm)
AC300	4.25	0.0037	8.02
AC500	25.94	0.0223	9.45
AC700	44.23	0.0305	10.29

The BET surface area, pore volume, and average pore diameter of the activated carbons available in the literature were analyzed and compared with the activated carbon produced. When Table 3 is analyzed, an increase in the specific surface area of activated carbons was observed at increasing pyrolysis temperatures, while a decrease in pore diameter was observed. In general, a higher pore size was observed when the activated carbons in the literature were compared with the activated carbon in this study. Therefore, although it showed a low surface area in tetracycline removal, it was observed that it showed a successful adsorption capacity thanks to its high pore size and active groups on the surface.

**Table 3:** Bet surface areas, pore diameters, and pore volumes of the activated carbons produced in this study

Material	Pyrolysis temperature (^o^C)	BET surface (m^2^/g)	Pore volume (cm^3^/g)	Average pore size (nm)	Ref
BC900	900	182.53	0.19	4.14	Shao et al. (2024)
AC	550	917	0.447	11–19 A	Yazidi et al. (2020)
TAC	-	1093	1.569	5.92	Sayğili and Güzel (2016)
SAC-3	900	1365.79	0.38	3.1	Wang et al. (2020)
BC	800	959.9	0.4	From 24.4 to 4.4	Jang et al. (2018)
Non-activated- SBA	750	2.33	0.0095	8.54	Yang et al. (2016)
Ferric-activated SBA		126.86	0.15518	4.44	
AC300	300	4.25	0.0037	8.02	This work
AC500	500	25.94	0.0223	9.45	
AC700	700	44.23	0.0305	10.29	

**Fig. 6:**
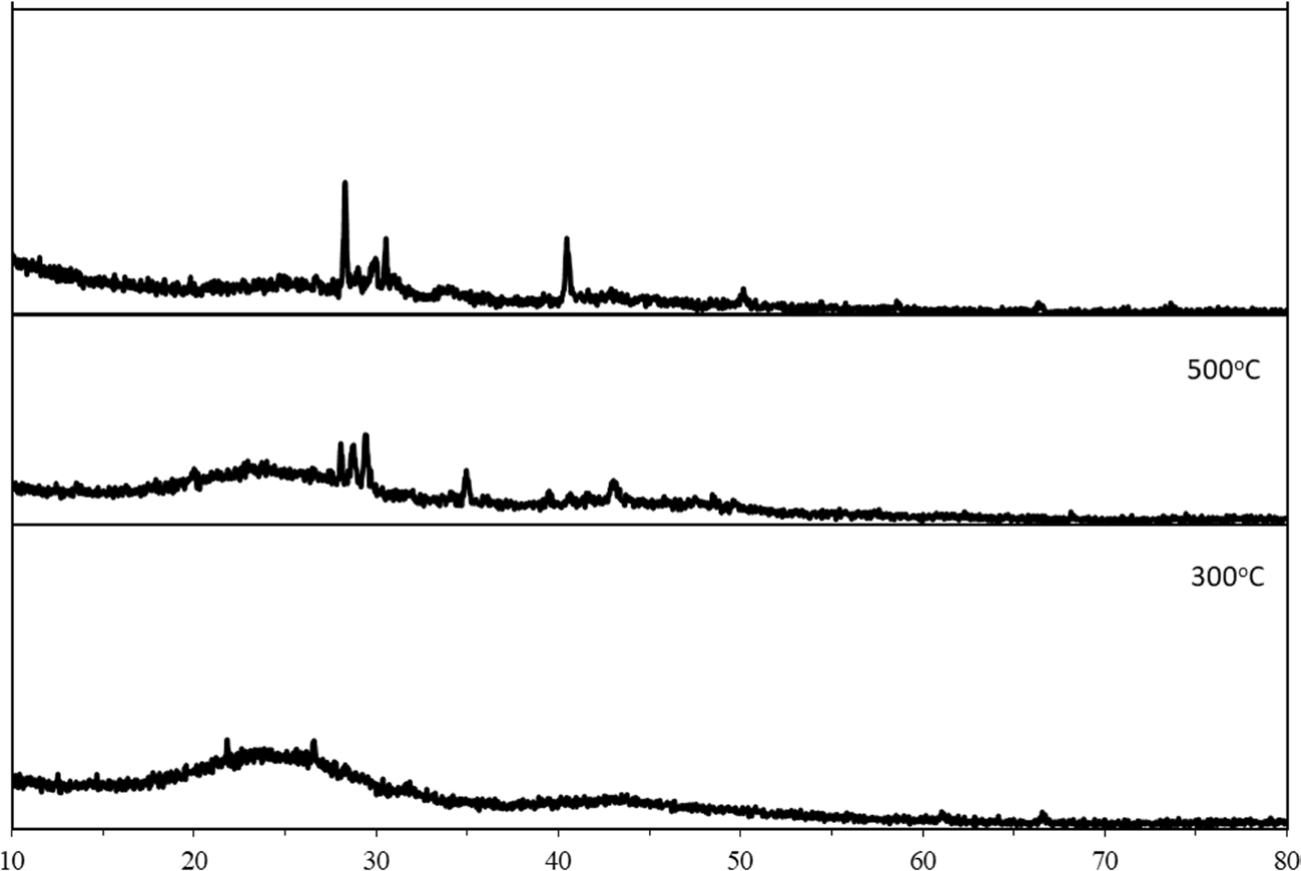
XRD patterns of activated carbons

According to the pore size distribution, the maximum pore size of the three samples appeared at 28–51 nm, indicating that the samples existed mainly in the form of medium pores. Besides, as can be seen from Fig. 6, grape marc waste at different pyrolysis temperatures has more characteristic XRD diffraction peaks at 10–80 °C, indicating the structural complexity of the activated carbon samples. It has been confirmed from the literature that the XRD diffraction peaks of the AC300 coincide well with the standard model of calcium oxalate. Among them, the diffraction peaks at 23.2°, 29.6°, 36.3°, 39.5°, 38.5°, and 43.8° represent the (− 110), (020), (− 202), (112), (− 411), (321) crystal planes of calcium oxalate. In addition, the XRD pattern of the sample shows almost no impurity peaks, confirming that sample AC300 is mainly composed of calcium oxalate. The location of the XRD diffraction peaks of AC500 and AC700 coincides well with the standard CaCO_3_ spectrum from the literature. Samples AC500 and AC700 had the highest peak at an XRD diffraction peak of 30.40°; this indicates that the AC500 and AC700 samples are mainly composed of calcium carbonate, and the higher and sharper the XRD peak force, the higher the crystallinity of the carbon samples. As a result, it can be seen that the XRD peak of AC700 is sharper among the three original activated carbons, also indicating that AC700 has the highest calcium carbonate crystallinity. At the same time, compared with the AC300 sample, the peak position of AC500 and AC700 samples changed greatly, indicating that different pyrolysis temperatures will affect the crystal structure of grape marc AC powder.

The X-ray diffraction pattern of each activated carbon sample and chestnut shell is shown in Fig. 6. When the X-ray diffraction patterns are examined, it is seen that the cellulose diffraction peaks seen between 16 and 22 in the grape marc samples used as raw material turn into almost a wide amorphous band structure in the obtained activated carbons and do not have the same crystal structure as the control sample. As the pyrolysis temperature increased, the crystalline structures derived from cellulose in the samples transformed into amorphous structures, and the crystalline peaks seen at the beginning were replaced by amorphous bands. On the other hand, X-ray diffraction patterns also provide information about the amount of cellulose the samples contain. The higher this ratio, the higher the crystal peak intensity of cellulose in the parallel direction. As a result, when the chestnut shell X-ray diffraction pattern is examined in detail, it can be seen that the cellulose crystal peak is more intense than the activated carbon samples.

**Fig. 7:**
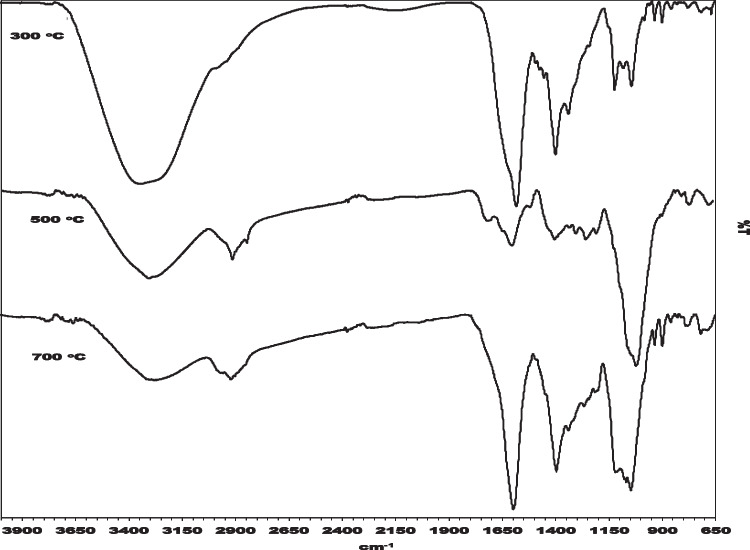
FTIR spectrum of activated carbons

AC300, AC500, and AC700 obtained in this research were examined by FTIR spectra and determined by chemical structure stability and thermal properties. Due to FTIR spectra, functional groups on the carbon surface were identified (Sarı et al. 2022). The FTIR spectra curves are shown in Fıgure 6. Carboxylic groups, lactans, and phenolic groups are acid surface oxides (Çalişkan et al. 2023). FTIR vibrations between 400 and 600 are because of the aromatic ring, while FTIR vibrations between 400 and 600 of ACS between 1350 and 1550 cm^−1^ are caused by lactonic groups (Arslanoğlu et al. 2020). The three characteristic infrared absorption peaks are the same, indicating that they have similar surface functional groups. The absorption peak at 871 cm^−1^ is located in the bending vibration zone within the C-H plane, possibly due to the flexural vibration within the C-H plane of the aromatic hydrocarbon (σ C-H). In another research, it was indicated that C-O vibrations show phenol, ether, and alcohol groups (Arslanoğlu et al. 2023). In this study, the absorption peak at 1056 cm^−1^ is due to the C-O telescopic vibration, and the adsorption peak at 1419 cm^−1^ is attributed to the C-H telescopic vibration in CH_2_ and CH_3_. There is a wide peak around 3453 cm^−1^ caused by the OH telescopic vibration. O–H vibrations indicated the moisture on the activated carbon surface (Arslanoğlu et al. 2023). At the same time, with the increase in pyrolysis temperature, the vibration intensity of hydroxyl stretching weakens. Different pyrolysis temperatures during pyrolysis result in slightly different infrared peaks. Previous studies have shown that the content of volatile organic compounds, dehydroxylation reactions, aromatics, and other components will increase during the pyrolysis process. The absorption peak area of the OCFG of AC300, AC500, and AC700 decreased sequentially, and the band intensity of O–H and aromatic C-O was significantly weakened, indicating that the OCFG in AC gradually cracked. The FTIR spectra of ACs show that some groups disappeared after the carbonization and activation steps of the biomass (Fig. 7).

### Effects of reaction time

**Fig. 8:**
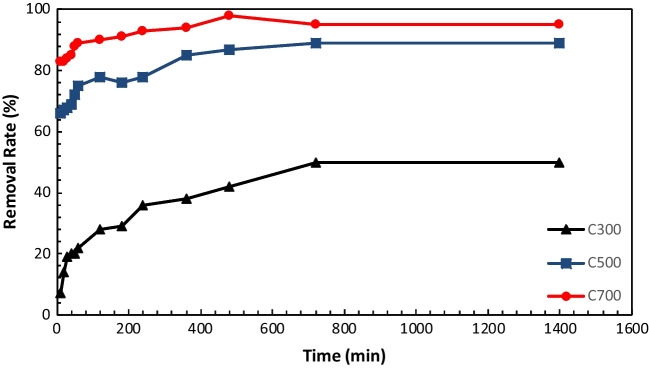
Effect of contact time on removal efficiency

The adsorption of tetracycline by ACs at different times was carried out by varying adsorption time from 0 to 1400 min. Figure 8 shows the effect of contact time on the adsorption of TC on ACs. Tetracycline removal was observed to increase gradually during the first 60 min. The reason for the initial adsorption rate is that TC can easily reach the available functional groups on the surface of the adsorbents (Mohubedu et al. 2019). When the adsorption time increases such as 8 or 12 h, the adsorption slows down with the filling of functional groups on the adsorption surface and finally stops. Because the adsorption surface and fewer adsorption sites were available, the removal efficiency remained constant with further increase in adsorption time (Zhang et al. 2019). The best adsorption efficiency was found to be the same time for all three ACs. Since the heterogeneous surface structure and properties of AC obtained at different pyrolysis temperatures are different, we think that the adsorption efficiency is also different for each batch.

### Effect of the dose of adsorbent

**Fig. 9:**
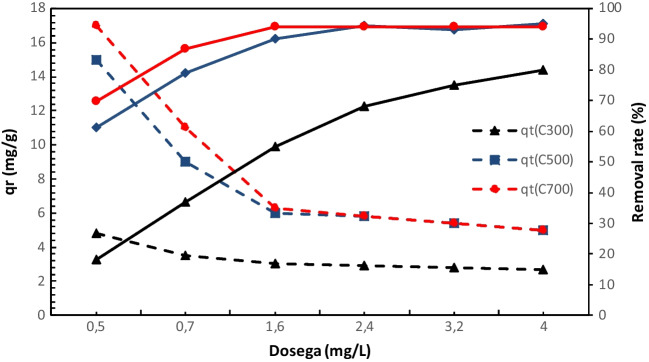
Effect of dosages on the absorption of tetracycline on ACs

The effect of dosages on the absorption of tetracycline on ACs (AC300, AC500, and AC700) was investigated in Fig. 9. The results showed that for all three adsorbents, the percentage of TC removal in wastewater increased with increasing the amount of adsorbent, whereas the increase in the amount of adsorbent can reduce the adsorption capacity of AC on TC. That is, as the amount of adsorbent increased, the adsorption capacity gradually decreased for all three activated carbons and approached each other. However, it was observed that this situation increased the adsorption removal efficiency. The reason for this decrease in adsorption capacity may be that the ratio of the number of TC molecules in solution to the vacant sites of the adsorbent decreases with increasing adsorbent weight, which reduces the possibility of interaction between adsorbent vacancies and TC molecules (Jannat Abadi et al. 2019). When the adsorbent dosage was increased from 0.5 to 1.5 g/L, the adsorption efficiency of AC500 increased from 61.29 to 90.55% and that of AC700 increased from 69.85 to 94.12%. Since both AC700 and AC500 TC removal was 94% when AC was taken at 1.5 g/L, the experiment was continued using adsorbent at this dosage. We see in many studies that the concentration of the adsorbent is one of the effective parameters in the adsorption process. For example, Hasan et al. reported in their study that by increasing the amount of CMH (adsorbent) at the optimum pH value, an increase in the ion loading capacity of the material was observed due to mass flow and that the solution concentration in the mass and the adsorbed adsorbent at the interface were effective in the process (Hasan et al. 2023).

### Effect of the initial pH of the solution

**Fig. 10:**
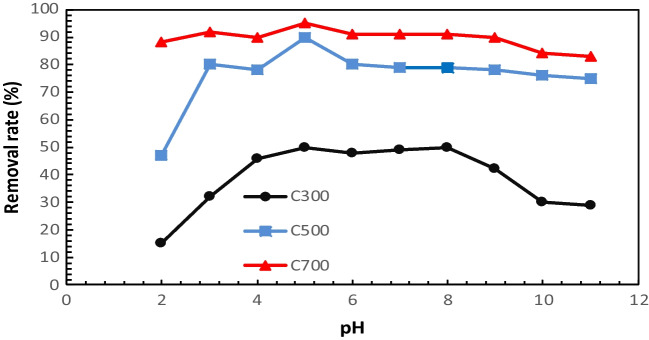
Effect of pH on the absorption of TC on ACs

The pH increase of the tetracycline species is a function of both the initial antibiotic concentration and the adsorption capacity of the adsorbent (Antón-Herrero et al. 2018). The pH of adsorption on the ACs for treatment wastewater might impact the surface charge and ionization degree (Suwunwong et al. 2020); therefore, pH is an important factor in adsorption. The effect of pH on the absorption of TC on ACS is given in Fig. 10. The results show that different pH values affected considerably the adsorption quality and TC absorption of activated carbons obtained at different temperatures. The removal of TC from wastewater when the initial pH range of 2.0–11.0 was investigated. An increase in pH is observed during the pyrolysis process because of the formation of carbonates during this process (Antón-Herrero et al. 2018), and it was observed that the adsorption started to decrease as the pH increased. TC absorption onto different ACs at pH is closely linked to the surface charge of chars. Tetracycline, an amphoteric molecule with multiple ionizable functional groups, can have a total charge of positive (pH 3.3 <), neutral (pH 3.3 < 7.8), or negative (pH > 7.68) (Zhao et al. 2011). TC absorption at pH 2 was lower than 10 mg/g. This is because when the pH of the solution is at its lowest (pH = 2.0), there is a large amount of H^+^ in the solution. This means that AC is electrostatically pushed by the protonation and the positively charged TC. In the adsorption of TCs with activated carbons, there is a saturation point where tetracycline can no longer be adsorbed because there is no negatively charged region left in the adsorbent for the binding of electrostatic forces between the negative charge of ACs and the positive charge of TCs (Chang et al. 2009). The pKa of TC is 3.3, 7.7, and 9.37, respectively. When the pKa values of TC adsorption on AC are above pH 3.3, the main ionic form is TCH^+2^ due to the loss of a proton from the phenolic diketone fraction and protonation of the dimethyl-ammonium group (Antón-Herrero et al. 2018). At pH = 4.0–7.0, the removal rate of AC from TC is high. AC300, AC500, and AC700 showed similar increasing and decreasing removal rates at different initial pHs. The best adsorption was obtained when the initial solution was close to neutral. pH affects the electrostatic interaction between AC and TC by affecting the charge carried by the two surfaces. It can be seen that electrostatic interaction is an important mechanism for AC to affect the removal of TC from water.

In many studies in the literature, the effect of pH on adsorption capacity has been mentioned (Hsu et al. 2023; Md. Munjur et al. 2020). For example, Salman et al. (2023b) reported that pH is the main parameter affecting adsorption capacity (Salman et al. 2023a).

### Adsorption kinetics

**Fig. 11:**
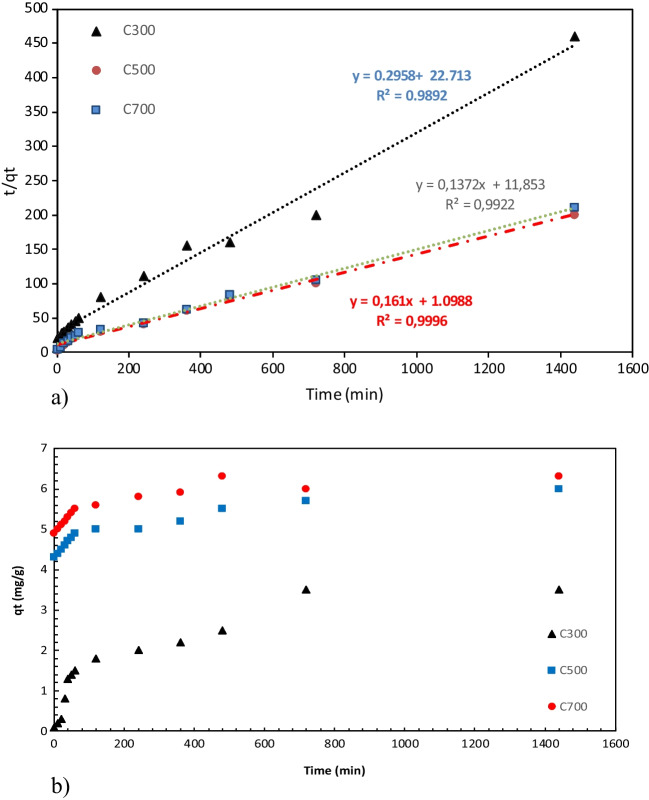
Kinetic plots of tetracycline on activated carbons

The kinetics show the adsorption of TC at different initial TC concentrations. The kinetic mechanism for TC adsorption on the adsorption surface plays an important role in obtaining information on the reaction pathway and the rate control mechanism of the exchange reaction (Suwunwong et al. 2020). In order to obtain more accurate results, we used the non-linear method and gave kinetic plots of TC on activated carbons in Fig. 11. Kinetic models of pseudo-first-order and pseudo-second-order are given in Table 4. As the solution temperature increased from 300 to 500 °C and 700 °C, the initial rate of TC adsorption increased from 0.006 to 0.013 g/mg min and 0.025 g/mg min. Temperature affects the adsorption kinetics as the rate constant k increases with increasing temperature and the equilibrium adsorption capacity (qe) increases with increasing temperature (Zhou et al. 2019). Another example of this can be seen in the work of Huang et al. It has been reported that an increase in adsorption capacity is observed with temperature increase (Huang et al. 2022a). The *R*^2^ values of the AC300, AC500, and AC700 quasi-secondary kinetic models are 0.9992, 0.9959, and 0.9991, respectively, whereas the *R*^2^ values of the quasi-first kinetic models are 0.8922, 0.1565, and 0.3863, respectively. Pseudo-quadratic kinetics for a chemical adsorption process is the limiting step (Chen et al. 2019). The pseudo-secondary kinetic model fits better than the pseudo-first kinetic model, indicating that the process takes place along the chemical adsorption (Unuabonah et al. 2007). Huang et al. reported in their studies that it showed heterogeneous adsorption with both physisorption and chemisorption pathways (Huang et al. 2023). Table 5 shows the kinetic parameters of tetracycline studies available in the literature.

**Table 4:** Model parameters on adsorption of tetracycline

		Pseudo-first kinetic model			Pseudo-secondary kinetic model		
Sample	q_e,exp_	q_e,cal_ (mg/g)	k_1_ (min/g)	*R*^2^	q_e,cal_ (mg/g)	k_2_ (g/mg min)	*R*^2^
C300	3.12	2.45	0.015	0.8612	3.19	0.002	0.9996
C500	5.70	4.15	0.391	0.3945	5.75	0.012	0.9989
C700	6.01	5.32	0.442	0.2457	6.19	0.021	0.9999

**Table 5:** Pseudo-first-order and secondary-order model results for tetracycline in the literature

		Pseudo-first kinetic model			Pseudo-secondary kinetic model			Ref
Sample	q_e,exp_	q_e,cal_ (mg/g)	k_1_ (min/g)	*R*^2^	q_e,cal_ (mg/g)	k_2_ (g/mg.min)	*R*^2^	
ZBAC	22.335	13.330	2.079	0.9696	18.812	0.7908	0.9571	Cai et al. (2019)
Lanthanum-modified diatomite	-	04.12	00.0110	0.479	57.9	0.0285	0.999	Li et al. (2015)
Biochar	5.21	5.16	9.40	0.995	5.24	6.49	0.999	Sun et al. (2021)

### Adsorption isotherms

Adsorption isotherms are used to determine the dynamic equations of adsorption systems and the maximum adsorption capacity (Suwunwong et al. 2020). The most common adsorption isotherm models are Langmuir and Freundlich (Arslanoğlu et al. 2023). In this study, isotherms were determined according to Langmuir and Freundlich’s isotherm models, and also these isotherms were used to determine the nature of TC adsorption on activated carbon at different temperatures. The adsorption capacity of ACs under various initial TC concentrations is given in Fig. 11. The relative parameters of each fitting isotherm are listed in Table 6. The fitting correlation coefficient *R*^2^ of Langmuir and Freundlich models of ACs obtained at different temperatures was found to be above 0.95. According to Langmuir isotherm, the maximum adsorption capacity was 17.88 mg/g at AC700, which can be explained by the effect of cation exchange (Jiang et al. 2015). The quadratic correlation values show that the points fit the two isotherm models well. The quadric correlation value and the maximum adsorption capacity were found in Langmuir isotherm, which indicates that it is more suitable for Langmuir isotherm. While the Langmuir isotherm refers to monolayer and homogeneous adsorption, the Freundlich isotherm assumes that adsorption occurs under inhomogeneous multilayer conditions (Li et al. 2020). Accordingly, we think that the absorption of TC occurs in the multilayer and monolayer adsorption layers. Similarly, there are many studies in the literature in which single-layer adsorption is appropriate, and one of them is the NTP-PAC adsorbent. NTP-PAC has been reported to be consistent with the pseudo-first-order kinetic model and exhibit heterogeneous adsorption (Huang et al. 2022b). Table 7 shows adsorption kinetics dimensionless constant separation factor. Table 8 shows the isotherm results of the studies on tetracycline available in the literature.

**Table 6:** Isotherm parameters on adsorption of tetracycline

	Langmuir			Freundlich		
Sample	q_m_ (mg/g)	K_L_ (L/mg)	*R*^2^	K_F_ [(mg/g)(mg/L)^n^]	1/n	*R*^2^
C300	6.56	0.25	0.9999	1.56	0.2923	0.9536
C500	14.01	0.399	0.9856	3.85	0.2765	0.9128
C700	17.88	0.457	0.9569	5.48	0.2846	0.9356

**Table 7:** Adsorption kinetics dimensionless constant separation factor

	*C*_*0*_ (mg/L)	*R*_*L*_		*C*_*0*_ (mg/L)	*R*_*L*_		*C*_*0*_ (mg/L)	*R*_*L*_
**AC300**	5.000	0.6281	**AC500**	5.000	0.3971	**AC700**	5.000	0.4213
	10.000	0.4400		10.000	0.2420		10.000	0.2607
	20.000	0.2750		20.000	0.1364		20.000	0.1474
	40.000	0.1573		40.000	0.0726		40.000	0.0792
	60.000	0.1100		60.000	0.0495		60.000	0.0539
	80.000	0.0847		80.000	0.0374		80.000	0.0407
	100.000	0.0693		100.000	0.0297		100.000	0.0330

**Table 8:** Isotherm results of studies on tetracycline adsorption in the literature

		Langmuir			Freundlich			Ref
Adsorbent	Adsorban	q_m_ (mg/g)	K_L_ (L/mg)	*R*^2^	K_F_ [(mg/g)(mg/L)^n^]	*n*	*R*^2^	
SWCNT	Oxytetracycline	737.5	0.101	89.7	107.5	2.14	80.8	Ncibi and Sillanpää (2015)
MFX	Tetracycline	3.75	3.30	0.99	6.24	1.21	0.98	Li et al. (2016)
Biochar	Tetracycline	71.54	0.08	0.994	15.92	1/n: 0.27	0.885	Sun et al. (2021)
Stevensite-based geofilter	Tetracycline	700.6	9	0.98	30.96	0.282	0.97	Fernández et al. (2018)

### Antibiotic adsorption mechanism

The hydrophobicity of activated carbon increases tetracycline adsorption. For example, Kulshrestha et al. suggested a hydrophobic mechanism on the surface in their study (Kulshrestha et al. 2004). The reason for this is that in tetracycline adsorption from water, the hydrophobicity of activated carbon will prevent water molecules from filling the pores, which will provide more active sites for tetracycline adsorption. In this study, the hydrophobicity increases according to the elemental composition, which increases the adsorption capacity.

When the pH is examined, we say that the most effective adsorption is obtained when the pH is neutral (pH 4.0–7.0). At pH 2, since the surface of both activated carbon and tetracycline is negative, they repel each other due to electrostatic interactions. Therefore, no effective adsorption can be observed. However, when the pH is close to neutral, the main ionic form is TCH + 2 for tetracycline due to the loss of a proton from the phenolic diketone fraction and protonation of the dimethyl-ammonium group, while the surface for activated carbon is negatively charged. In the pH 4–7 range, the adsorption was observed as the most effective pH due to electrostatic interactions between the positively charged surface of tetracycline and the negatively charged surface of activated carbon. Similar results were also observed in a study by Zhao et al. They reported that kaolinite adsorbed tetracycline most efficiently at pH 3–6 (Zhao et al. 2011). The reason for this increase in adsorption is thought to be the surface bridging mechanism in pH-neutral and alkaline conditions as stated in Wang et al. (2008).

**Fig. 12:**
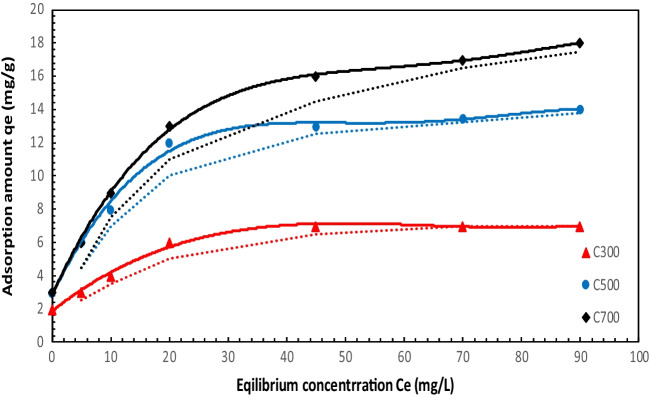
Adsorption isotherms fitting of TC on ACs

When N_2_ adsorption/desorption isotherms were analyzed, it was found that the highest BET surface area was 44.23 m^2^/g for AC700. When the literature is examined (Table 9), this value is quite low. However, when adsorption studies were examined, it was determined that the activated carbon produced from grape marc showed a good adsorption capacity thanks to its surface functional groups (Fig. 12).

**Table 9:** Similar studies in the literature

Activated carbon	Initial tetracycline concentration	BET surface area (m^2^/g)	Adsorption capacity	References
Activated carbon	5000 ng/L	852.94	99.4%	Zhang et al. (2016)
Activated carbon from coconut shells	10 10 µg/L − 1	1100	96–100%	Choi et al. (2008)
Activated carbon from apricot peel	100–200 ve 150 mg/L	307.6	308.33 mg g–1	Marzbali et al. (2016)
Activated carbon from crab shell (K-CSB)	100 mg/L	181.79	380.92, 96.98%	Sun et al. (2023)
Granular activated carbon	1700 mg/L	1059.011	17.02 mg/g	Li et al. (2022)
Activated carbon (AC) derived from invasive *Sargassum (sp)*	200 mg/L	1005 ± 163 m^2^ g^−1^	48.9%, 579.69 mg g^−1^	Francoeur et al. (2022)
Activated carbon from rice straw	0.5 –32 mg/L)	27.66	92.8–96.7% 14.16 mg/gg	Wang et al. (2017)
Activated carbon derived from alfalfa	20 mg/L	796.50	302.37 mg/g	Jang and Kan (2019b)
Activated carbon from willow branches	20 mg/L	3342 m^2^/g	1300 mg/g	Yang et al. (2019)
Activated carbon from straw	100 mg/L	1102	372	Jang and Kan (2019a)

Ibn Ferjani et al. mentioned the remarkable properties of pyrolysis temperature on biochar in their study. According to the study, they reported that hemicellulose compounds or alkali elements were degraded at 210–290 °C. At 320 °C, they reported that cellulose decomposed and at 500 °C, lignin started to decompose. At 700 °C, they reported that the material had a carbon content of 30.9%, that is, the carbon percentage increased. In summary, we understand that the increase in pyrolysis temperatures affects the functional groups on the surface, and in this context, the surface of the biochar is shaped. They presented data on the efficiency of the activated carbons obtained in the experiment carried out in the temperature range of 300–700 °C. In light of the data presented, they reported that the activated carbon obtained at 300 °C temperature has lower carbon content and higher mineral content compared to the activated carbon obtained at 700 °C temperature (Ibn Ferjani et al. 2019). In this study, the FTIR analysis was used to make sense of the surface functional groups of the activated carbons obtained and to examine the difference in adsorption capacity. The FTIR analysis is one of the analyses applied to examine the surface functional groups of porous carbons such as activated carbon. In this study, the peak decrease of AC300 compared to AC700 in the FTIR analysis given in Fig. 7 was predicted to occur after both carbonization and activation stages. On the other hand, when Table 1 is examined, it is observed that while AC300 content is 42.5 wt%, it increases to 51.63% in AC700. This shows that the aromatic compounds on the surface of the material are removed and more active sites are provided.

**Fig. 13:**
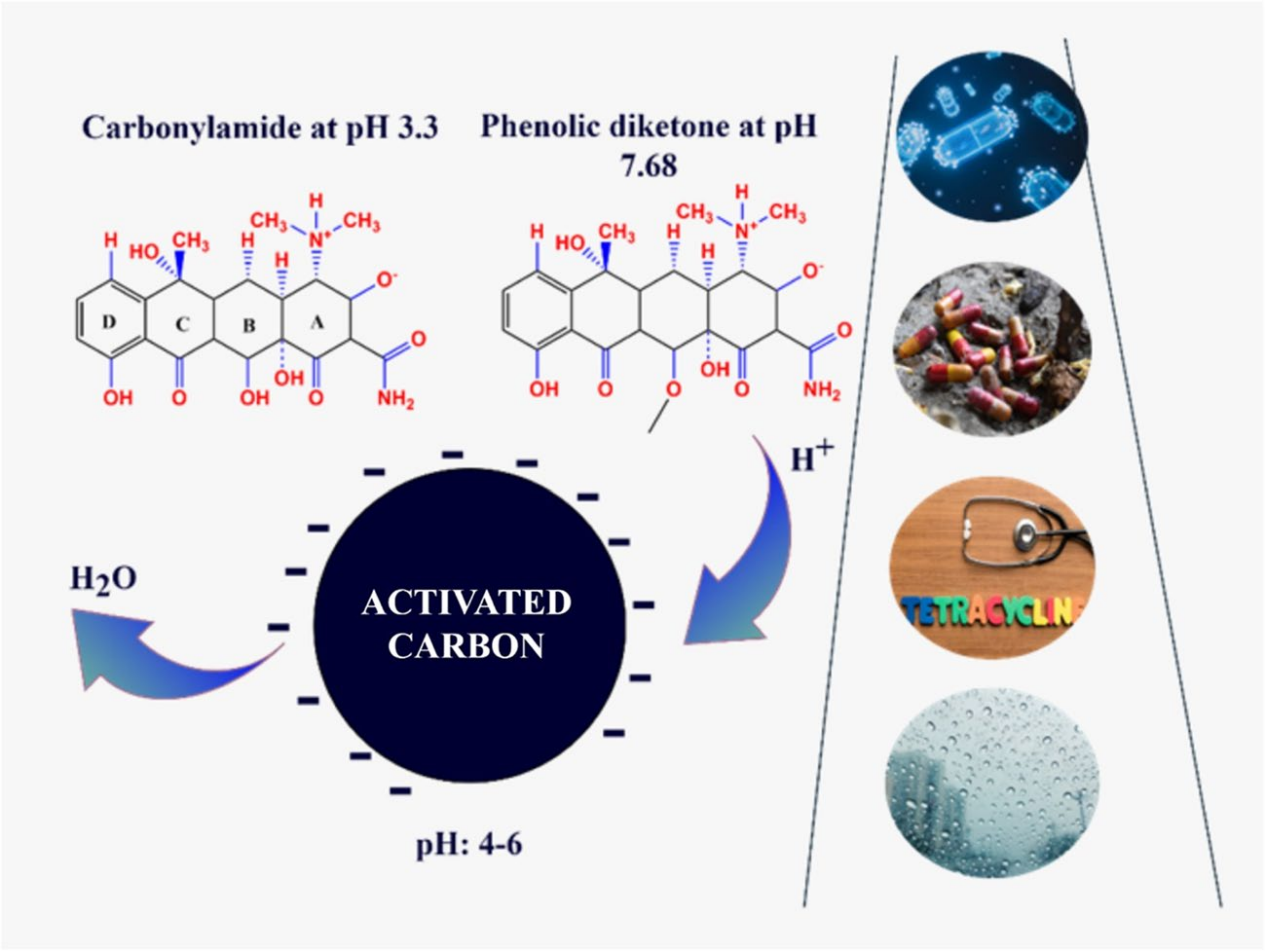
Adsorption mechanism

Figure 13 shows that the adsorption capacity decreases gradually with the increase of ion concentration, indicating that the adsorption capacity is affected by the ionic strength of the solution. The larger the ionic strength is, the smaller the adsorption capacity is. In addition, the adsorption capacity of TC on AC is also affected by the pore size of the AC surface, hydrogen bond, and π-π interaction.

### Adsorption studies in the literature

In a study, they used powdered activated carbon (PAC) they produced for the removal of 28 types of antibiotics, including tetracycline (TC), from water. The specific surface area of PAC was 852.94 m^2^/g, and the iodine adsorption value was 903 mg/L. They kept the initial concentration of 5000 ng/L for each antibiotic. They carried out the studies with PAC dosage in the dosage range of 5–50 mg/L. They reported that the best removal for TC was achieved at 50 mg/L adsorbent dosage with 99.4% efficiency (Zhang et al. 2016).

Choi et al. investigated the adsorption of TC and sulfonamide (SA) by activated carbon powdered from coconut. They stated that the phenolic components of TC were responsible for its high adsorption capacity. They reported that the metal and metal oxide components on the surface of the activated carbon they produced provided high adsorption capacity for TC in complex formation. The activated carbon has a surface area of 1100 m^2^ g^−1^. A total of 96–100% TC removal dosed at 0.7 mg L^−1^ activated carbon was reported (Choi et al. 2008).

They named the activated carbons activated with three different chemical agents, KOH, H3PO4, and KMnO4 as K-CSB, P-CSB, and M-CSB, respectively. They obtained higher TC adsorption capacity by activating tetracycline adsorption capacity with a potassium hydroxide chemical agent. At 800 °C pyrolysis temperature, they obtained maximum bet surface area. They reported that the increase in temperature increased the porosity on the surface due to the loss of organic matter. After chemical activation, they reported that this value increased even more and the surface area was 1095.14 m^2^/g. They reported that at high temperatures, potassium hydroxide reacts with carbon and produces K_2_CO_3_, K_2_O, and H_2_, and at the end of the reaction, metallic K volatilizes and forms arak atman. In this way, they think that they achieved the highest surface area with potassium hydroxide (Sun et al. 2023).

In general, it has been observed that efficient TC removal is achieved thanks to high surface areas. In this context, reviews from the literature are given in Table 9.

## Conclusıon

Tetracycline is one of the most widely used antibiotics. However, its presence in wastewater is very harmful because it increases antibiotic resistance and is toxic. Therefore, in this study, activated carbon was produced for tetracycline removal. In this study, activated carbons were obtained from grape marc at different pyrolysis temperatures. It was observed that the BET surface area of the obtained activated carbons increased from 4.5 to 44.23 at increasing temperatures, while the pore size of the activated carbons increased from 8.02 to 10.29 when the temperature increased from 300 to 700 °C. When the adsorption isotherms and kinetics of activated carbons were analyzed, it was found that the most suitable isotherm was Langmuir isotherm and it was calculated as 6.98 mg/g for AC300, 15.25 mg/g for AC500, and 17.38 mg/g for AC700 according to semi-secondary kinetics. In the kinetics, TC removal was observed for all three activated carbons, initially increasing and gradually continuing at a constant rate. When SEM surface images of activated carbons were analyzed, it was observed that a more porous structure was formed on the surface of activated carbons at increasing temperatures. The physical and chemical properties of activated carbons were analyzed by FTIR, XRD, and BET. According to these results, pyrolysis temperature had a significant effect on adsorption capacity. When the literature was examined, it was observed that the activated carbon in this study had a higher pore diameter than the activated carbon in this study, although the existing studies had a very high surface area. Although it has a low surface area, effective tetracycline removal was achieved thanks to both the active sites on the surface and the pore size. As a result, TC adsorption on the surface of activated carbons was found to be successful. In the following stages of the study, the obtained activated carbons can be studied for tetracycline removal from wastewater. Gas adsorption, heavy metal removal, and dyestuff removal can also be investigated.

## Data Availability

The data that support the findings of this study are available from the corresponding author upon reasonable request.
